# Field Dissipation and Storage Stability of Glufosinate Ammonium and Its Metabolites in Soil

**DOI:** 10.1155/2014/256091

**Published:** 2014-10-13

**Authors:** Yun Zhang, Kai Wang, Junxue Wu, Hongyan Zhang

**Affiliations:** College of Science, China Agricultural University, Beijing 100193, China

## Abstract

A simple analytical method was developed to measure concentrations of glufosinate ammonium and its metabolites, 3-methylphosphinico-propionic acid (MPP) and 2-methylphosphinico-acetic acid (MPA), in field soil samples. To determine the minimum quantification limit, samples were spiked at different levels (0.1, 0.5, and 1.0 mg/kg). Soil samples were extracted with ammonium hydroxide solution 5% (v/v), concentrated, and reacted with trimethyl orthoacetate (TMOA) in the presence of acetic acid for derivatization. The derivatives were quantified by gas chromatography (GC) using a flame photometric detector (FPD). The linear correlation coefficients of glufosinate ammonium, MPP, and MPA in soil were 0.991, 0.999, and 0.999, respectively. The recoveries of this method for glufosinate ammonium, MPP, and MPA in soil were 77.2–95.5%, 98.3–100.3%, and 99.3–99.6% with relative standard deviations (RSD) of 1.8–4.1%, 0.4–1.4%, and 1.3–2.0%, respectively. Glufosinate ammonium dissipated rapidly in soil to MPA in hours and gradually degraded to MPP. The half-life of glufosinate ammonium degradation in soil was 2.30–2.93 days in an open field. In soil samples stored at −20°C glufosinate ammonium was stable for two months. The results of this study should provide guidance for the safe application of the herbicide glufosinate ammonium to agricultural products and the environment.

## 1. Introduction

Glufosinate ammonium, ammonium (3-amino-3-carboxypropyl)methyl phosphinate, is a broad-spectrum contact herbicide and a crop desiccant (dries crops before harvest) originally developed by AgrEvo. Glufosinate, also known as phosphinothricin, is a naturally occurring phytotoxin that was first isolated from the bacteria, Streptomyces viridochromogenes [1–3]. Glufosinate ammonium is used worldwide to control a broad range of both annual and perennial broadleaf weeds in fruit orchards, vineyards, rubber and oil palm plantations, ornamental trees and bushes, noncrop land, and preemergence in vegetables [4, 5]. In addition, glufosinate ammonium is used as a desiccant in potatoes, sunflowers, and so forth [6, 7]. In soil, glufosinate ammonium is mainly degraded to 3-methylphosphinico-propionic acid (MPP), which may undergo further degradation to 2-methylphosphinico-acetic acid (MPA). The most important factor affecting glufosinate ammonium degradation in soil is microorganisms, while other factors such as temperature, light, and rainfall may increase the degradation rate [1, 2, 8–11]. Glufosinate ammonium is soluble in water (>500 g L^−1^ at pH 5–9, 20°C) and stable to light and hydrolysis at pH 5, 7, and 9 [7].

Simultaneous determination of glufosinate ammonium and its metabolites is difficult due to their high polarity, low volatility, high aqueous solubility, and lack of either UV chromophore or fluorescence [12–14]. Therefore, these compounds require derivatization before analysis by gas chromatography (GC) or liquid chromatography (LC) can be performed.

Prior to our studies, the detection of glyphosate, aminomethylphosphonic acid (AMPA), and glufosinate by capillary electrophoresis with indirect fluorescence detection was reported by Chang and Liao [15]. In 1996, Kataoka et al. developed a method for the determination of glyphosate and glufosinate in river water, soil, and carrot samples by GC using a flame photometric detector (FPD) after derivatization [16]. Their derivatization method involved the use of isopropyl chloroformate (isoPCF) and diazomethane, reagents that are potentially explosive and carcinogenic thereby limiting the use of this method. Qian et al. used 4-chloro-3,5-dinitrobenzotrifluoride (CNBF), which may react with primary or secondary amines in the sample, to derivatize glufosinate in maize samples for quantification by LC [17]. Sancho et al. analyzed glufosinate, glyphosate, and AMPA concentrations in water samples using 9-fluorenylmethylchloroformate (FMOC-Cl) for precolumn derivatization followed by LC with fluorescence detection (FLD) [18]. However, FMOC-Cl derivatization of MPP and MPA present in the sample was unsuccessful, and no response values were found by LC-FLD. A superior method was reported by Tsuji et al. for the simultaneous determination of glufosinate, its metabolite, and glyphosate present in brown rice, whole wheat, cabbage, tomato and onion with acetic acid and trimethyl orthoacetate (TMOA) [19]. Stalikas and Pilidis also used TMOA as a derivatization agent for the determination of pesticides containing amino acid groups by GC with mass-selective detection (MSD) [20]. In addition, Tseng et al. derivatized glyphosate, glufosinate, and their major metabolites AMPA and 3-MPPA with TMOA and simultaneously determined their concentrations in samples of rice and soybean sprouts using GC-PFPD [11]. Royer et al. also used this derivatization method to detect glufosinate ammonium and its metabolites MPP and MPA in water by GC with tandem mass spectrometry [3]. To our knowledge, there are no reports of a method to simultaneously detect glufosinate ammonium and its metabolites MPP and MPA and determine their environmental fate in field soil samples.

The aim of this study was to develop an accurate and cost-effective GC-FPD method to evaluate the dissipation of glufosinate ammonium and its metabolites after treatment in soil collected from open field trials and their storage stability at −20°C.

## 2. Materials and Methods

### 2.1. Materials

The analytical standards for glufosinate ammonium (99.2%), MPP (97.9%), and MPA (99.4%) were obtained from Beijing Perfect Technology Co., Ltd. (Beijing, China). The glufosinate ammonium formulation (200 g L^−1^ aqueous solution (AS)) was purchased from Hebei Veyong Bio-Chemical Co. Ltd. (Hebei province, China). Standard solutions of glufosinate ammonium, MPP, and MPA were prepared with methanol (1.0 g L^−1^). Working standard solutions for calibration were prepared by dilution with methanol to concentrations of 0.05 to 5.0 mg L^−1^.

Trimethyl orthoacetate (analytical reagent grade) was purchased from Sigma-Aldrich (USA). Acetic acid and ammonium hydroxide (both analytical reagent) were purchased from Sinopharm Group Chemicals Co., Ltd. and Beijing Chemical Reagent Company (Beijing, China), respectively. Ethyl acetate (HPLC-grade) was purchased from Fisher Scientific (Fair Lawn, NJ, USA). Water (HPLC-grade) was prepared using a Milli-Q water purification system (Millipore, USA).

### 2.2. Open Field Dissipation Experiments

The field trials for the dissipation experiments were conducted at two different locations, Xingcheng (Liaoning province, northeast China, monsoon climate) and Zibo (Shandong province, eastern China, warm temperate climate) from June to September in 2013. Field trials were carried out from July 29 until September 26 in Liaoning province and from June 30 to August 11 in Shandong province.

The field trials comprised two treatments: the first with glufosinate ammonium and the second, the control, with no treatment. Each treatment was replicated on three field plots, each plot consisting of an area of 30 m^2^. For the dissipation experiment, glufosinate ammonium (AS, 200 g L^−1^) was sprayed on the surface of the soil at a dosage of 1350 g a.i. ha^−1^. Soil samples were collected from the three replicate plots from 0 (2 h), 1, 2, 3, 5, 7, 10, 14, 21, 28, and 42 days after spraying. The control test was conducted simultaneously on three replicate field plots without application of glufosinate ammonium. Approximately 1 kg of soil was randomly collected to a depth of 0–10 cm in each plot. The soil samples were sifted through a one mm sieve, thoroughly mixed, and then stored at −20°C until analyzed.

### 2.3. Storage Stability Experiment

To investigate the storage stability of glufosinate ammonium containing soil samples, glufosinate ammonium (AS, 200 g L^−1^) was sprayed onto the surface of the soil at a dosage of 1350 g a.i. ha^−1^. Soil samples were randomly collected to a depth of 0–10 cm 2 h after spraying. After the soil samples were thoroughly mixed, they were transferred into eight sealed plastic bags, each bag contained 200 g of soil, and then stored at −20°C. For the storage stability experiment, these samples were analyzed on 0, 1, 3, 7, 14, 21, 30, and 60 days after storage.

### 2.4. Analytical Method

#### 2.4.1. Sample Preparation

A 5.0 g portion of the soil sample was weighed into a 100 mL Erlenmeyer flask and extracted with 50 mL of ammonium hydroxide 5% (v/v) by shaking for 1.5 h on an oscillator at 160 rpm. The extract was transferred to a 50 mL centrifuge tube and then centrifuged at 3800 rpm for 10 min. A 20 mL portion of the supernatant was then transferred to a 100 mL round bottom flask and evaporated to dryness at 60°C under reduced pressure using a rotary evaporator. Acetic acid (0.75 mL) and TMOA (1.5 mL) were then added to the residue and the mixture was sonicated at ultrasonic frequency 40 KHz for 5 min. The solution was then heated in a water bath at 95°C for 1.5 h to complete the derivatization reaction and evaporated to dryness at 55°C using a rotary evaporator. The concentrated derivatives were dissolved in ethyl acetate (2.0 mL) and filtered through a 0.22 *μ*m polytetrafluoroethylene (PTFE) membrane filter prior to analysis by GC-FPD.

#### 2.4.2. GC-FPD Analysis

The analysis was carried out with a SHIMADZU 2010 gas chromatography equipped with a flame photometric detector (GC-FPD). Separation of the analytes was achieved using a RXI-17 fused silica capillary column (30 m × 0.25 mm i.d., 0.25 *μ*m film thickness) with a standard method (80°C for 1.5 min, gradient of 30°C min^−1^, hold at 175°C for 2 min, gradient of 10°C min^−1^, hold at 185°C for 1 min, gradient of 30°C min^−1^, hold at 250°C for 10 min). The injection volume was 2 *μ*L in splitless mode. The injector and detector temperature were maintained at 220°C and 270°C, respectively. The carrier gas was high purity nitrogen (99.999%), which was set to a constant linear velocity with an initial flow rate of 1.0 mL min^−1^.

#### 2.4.3. Recovery Experiment

Soil samples were spiked with glufosinate ammonium, MPP, and MPA (0.1, 0.5, and 1.0 mg kg^−1^ each) and left to stand for 1 h. Five independent determinations were made for every fortification level. The spiked soil samples were extracted and derivatized according to the procedure described in the section: Sample Preparation. Blank soil samples were analyzed to verify the matrix effect.

## 3. Results and Discussion

### 3.1. Derivatization

**Figure 1:**
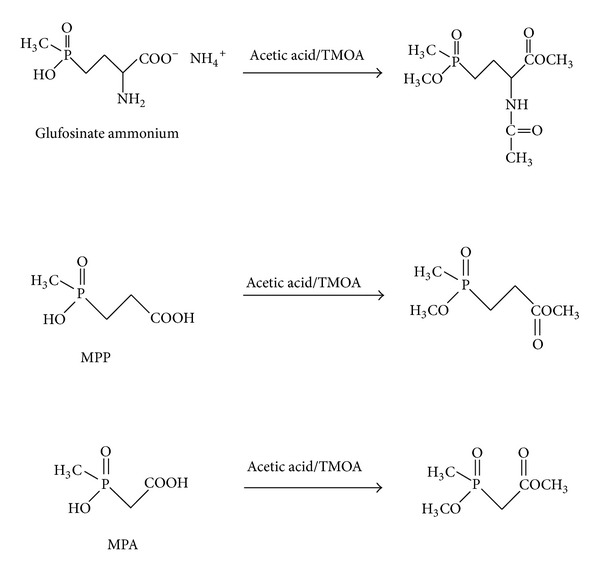
Derivatization reaction of glufosinate ammonium, MPP, and MPA with TMOA.

Glufosinate ammonium, MPP, and MPA were derivatized by treatment with TMOA in the presence of acetic acid; derivatization reactions are shown in Figure 1. Upon treatment with TMOA and acetic acid, the hydroxyl and amino groups of glufosinate ammonium, MPP, and MPA were acetylated and the carboxylic groups were converted to methyl esters [12, 20].

**Figure 2:**
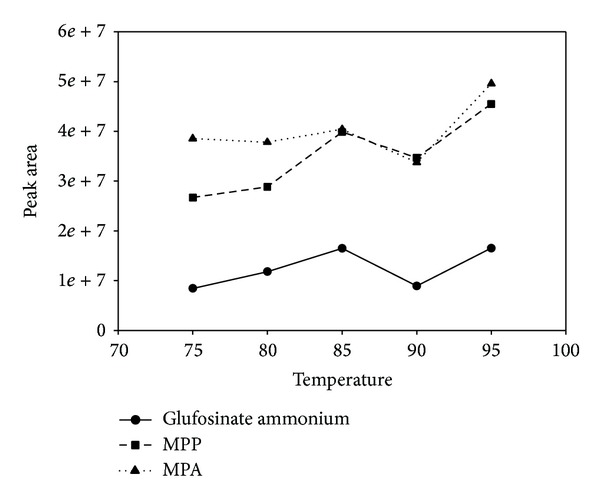
Optimization of derivatization temperature for glufosinate ammonium, MPP, and MPA.

**Figure 3:**
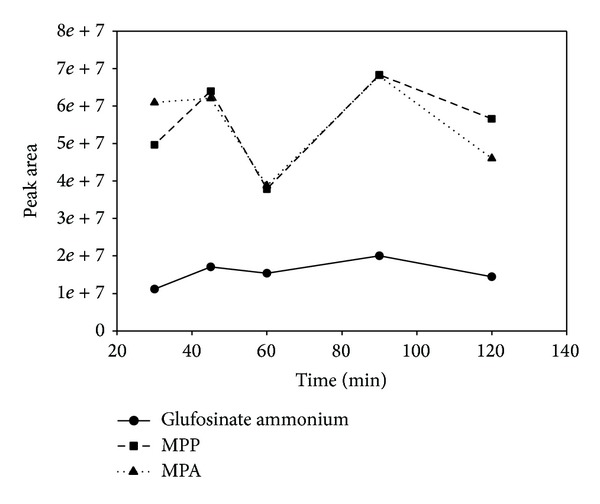
Optimization of derivatization time for glufosinate ammonium, MPP, and MPA.

To ensure the derivatization reaction proceeded to completion, the reaction temperature and time were optimized. TMOA was added according to Tseng's [11] conditions without further optimization. A mixture of the standard solutions (2 mL total volume) containing glufosinate ammonium, MPP, and MPA (2.0 ng of each) was reacted with TMOA and acetic acid at the following temperatures: 75°C, 80°C, 85°C, 90°C, and 95°C for 90 min. The results are shown in Figure 2. However, elevating the temperature from 75°C to 95°C did not dramatically increase the rate of reaction. The fastest reaction occurred at 95°C; higher temperatures were not tested because reaction temperatures close to the boiling point of water were determined to be operationally unsafe. After determining 95°C as the optimal temperature, the following reaction times were investigated: 30, 45, 60, 90, and 120 min. The results, shown in Figure 3, indicate that the best conversion was achieved after 90 min. In conclusion, the optimum temperature and time for the derivatization reaction were found to be 95°C and 90 min, respectively.

**Figure 4:**
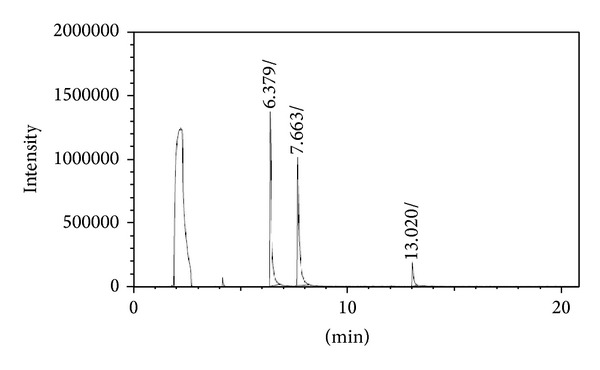
Gas chromatogram of the derivatives of glufosinate ammonium (1 mg L^−1^), MPP (1 mg L^−1^), and MPA (1 mg L^−1^).

MPA, MPP, and glufosinate ammonium derivatives were analyzed by GC-FPD using the conditions described (see* GC-FPD analysis*) and showed good response and separation.  Figure 4 shows a representative example of a gas chromatogram for the derivatized compounds, each injected at a concentration of 1.0 mg L^−1^.

### 3.2. Optimization of Extraction

The solubility of glufosinate ammonium in water is 1370 g L^−1^ (22°C) [19]; however it is essentially insoluble in the majority of organic solvents. As a result, an aqueous solution was chosen for extraction. Druart et al. used water for the extraction of glufosinate, glyphosate, and AMPA from soil [6]. In addition, Royer et al. extracted and purified glufosinate ammonium and its metabolites in water with an anion-exchange column; the analytes were washed with ultrapure water and eluted with 50% formic acid [3]. The acidity or basicity of a solution, measured by its pH value, is an important factor influencing the ability to extract polar compounds. Therefore, pure water, water with 0.1% acetic acid, and water with 0.1% ammonium hydroxide were tested to extract glufosinate ammonium, MPP, and MPA from soil samples. The extraction capacity of the alkaline solution was found to be superior to the others by comparison. Therefore, ammonium hydroxide was selected and the concentration of the ammonium hydroxide solution was investigated further.

**Figure 5:**
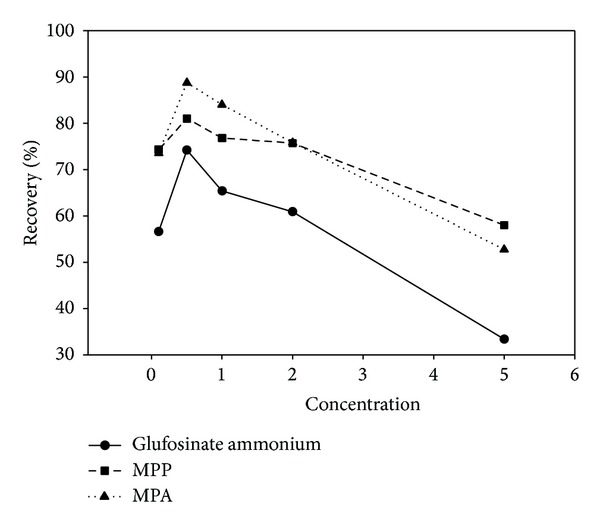
Optimization of the concentration of ammonia in the extraction solution.

Ammonium hydroxide solutions were investigated at concentrations of 0.1, 0.5, 1, 2, and 5% (v/v) and the results are shown in Figure 5. The recoveries of glufosinate ammonium and its metabolites increased with the concentration of the ammonia solution until 0.5%; after that the recovery decreased with increasing concentration. Therefore, ammonia hydroxide solution 0.5% (v/v) was optimal for the extraction protocol.

**Figure 6:**
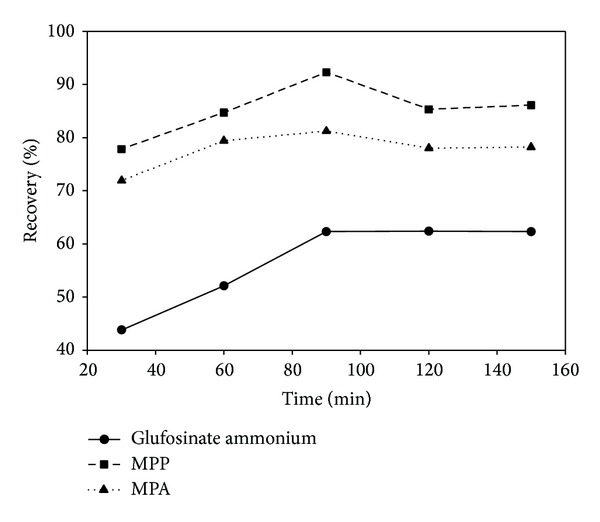
Optimization of oscillation time.

Finally, the oscillation time was optimized for extractions using ammonium hydroxide solution 0.5% (v/v) with an oscillator at 160 rpm. Soil samples were shaken for 30, 60, 90, 120, and 150 min; the extraction recoveries are shown in Figure 6. Results show that the recoveries of the compounds reached a maximum in less than 90 min and remained almost unchanged for samples shaken for a longer duration. The optimum shaking time of 90 min was used for all procedures.

### 3.3. Method Validation

#### 3.3.1. Calibration Curves, LOD, and LOQ

Linear calibration curves were obtained for glufosinate ammonium, MPP, and MPA by plotting the average peak area versus the concentration. The seven point calibration curves for the three compounds tested varied in a range from 0.05 to 5.0 mg L^−1^ in soil matrix. The calibration curves showed good linearity with correlation coefficients (*r*) between 0.991 and 0.999. The calibration curves were used to calculate the concentrations of glufosinate ammonium and its metabolites in soil. The limit of detection (LOD) values for glufosinate ammonium, MPP, and MPA were 0.01, 0.005, and 0.005 mg L^−1^, respectively, which is lower than the LOD value reported by Tseng et al. for glufosinate and 3-MPPA [11]. The limit of quantification (LOQ) values for glufosinate ammonium, MPP, and MPA were 0.05, 0.02, and 0.02 mg kg^−1^, respectively.

#### 3.3.2. Accuracy and Precision

Recovery is a significant challenge when attempting to quantify trace levels of an analyte from a complex matrix. To evaluate the accuracy and precision, soil samples were spiked with the standard solutions of glufosinate ammonium, MPP, and MPA at 0.1, 0.5, and 1.0 mg kg^−1^. Five replicates were performed for each fortification level. The results are shown in Table 1.

The fortified recoveries ranged from 77.2% to 100.3%. The precision of the method calculated by the relative standard deviation (RSD) ranged from 0.4% to 4.1%. The recovery and precision results were acceptable and met the acceptability criteria of the Residues Analysis Quality Control Guide (General Administration of Quarantine of the People's Republic of China 2002).

### 3.4. Degradation Dynamics of Glufosinate Ammonium in Soil

**Figure 7:**
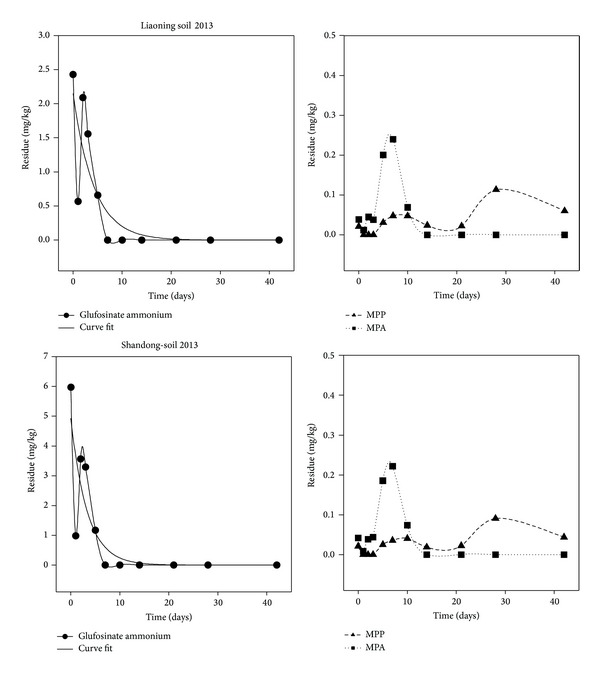
Dissipation of glufosinate ammonium in soil in Liaoning and Shandong in 2013.

The dissipation samples were analyzed and the results were plotted as a graph of glufosinate ammonium concentration versus time. The dissipation curve for glufosinate ammonium in soil displays first order kinetics as shown in Figure 7. The initial concentrations of glufosinate ammonium were 2.43 and 5.97 mg kg^−1^ at 2 h after treatment for the field trials in Liaoning and Shandong province, respectively. A gradual decrease in the glufosinate ammonium concentration in the treated soil was observed as a function of time after application. The dissipation equation for glufosinate ammonium concentrations in soil was *C* = 2.1477*e* ^−0.2366*t*^ with a correlation coefficient of 0.8501 and a half-life of 2.93 days in Liaoning province and *C* = 4.9316*e* ^−0.3017*t*^ with a correlation coefficient of 0.8553 and half-life of 2.30 days in Shandong province. There was no significant difference between the half-life of glufosinate ammonium in soil at two locations. These results indicate that glufosinate ammonium degradation in soil is not affected by the weather or the soil type, pH, and water content.

As expected, an increase in the concentration of the metabolite MPA occurred from 2 h to seven days after glufosinate ammonium treatment in soil samples from Liaoning and Shandong provinces. The peak concentration was measured on the seventh day; for Liaoning province it was 0.24 mg kg^−1^ and for Shandong province it was 0.22 mg kg^−1^. After the seventh day, the concentration of MPA gradually and continuously dissipated until it could no longer be detected on the fourteenth day. Unlike metabolite MPA, metabolite MPP could not be detected until the fifth day after application of glufosinate ammonium to the open field. The peak concentration of MPP was achieved on the twenty-eighth day; for Liaoning province it was 0.11 mg kg^−1^ and for Shandong province it was 0.09 mg kg^−1^. Although the initial concentrations of glufosinate ammonium were 2.43 and 5.97 mg kg^−1^ in Liaoning and Shandong, respectively, the peak concentrations for the metabolites MPA and MPP are similar. This observation may indicate that glufosinate ammonium can degrade in soil to both MPP and MPA.

### 3.5. Storage Stability of Glufosinate Ammonium in Soil

**Figure 8:**
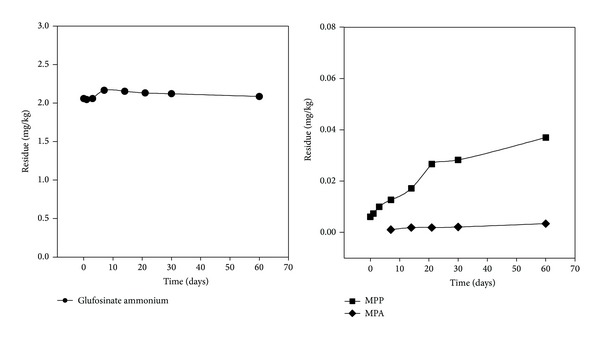
Storage stability of glufosinate ammonium in soil samples at −20°C.

Figure 8 shows the storage stability data for glufosinate ammonium in soil samples. These results indicate that glufosinate ammonium in soil samples are stable for 60 days after spraying when stored at −20°C. Therefore, this method is reliable since soil samples can be stored at −20°C for up to two months before being analyzed and still provide accurate results.

## 4. Conclusion

Glufosinate ammonium, MPP, and MPA in soil were extracted with ammonium hydroxide solution 5% (v/v) and derivatized with TMOA under the optimized conditions before being analyzed by GC-FPD. In comparison to the pretreatment methods previously discussed, this method is environmentally friendly, inexpensive, and easy to execute. The method used for the extraction and quantification of glufosinate, MPP, and MPA residues was found to be qualitatively and quantitatively accurate. Glufosinate ammonium dissipated with a half-life of 2.30–2.93 days in soil samples from two different locations in the northeast and east of China. Glufosinate ammonium in soil samples stored at −20°C was stable for 2 months. The results of this study should provide guidance for the safe application of glufosinate ammonium to agricultural products and environment.

## Figures and Tables

**Table 1:** Average recovery and RSD of glufosinate ammonium, MPP, and MPA in soil matrix.

Target compound	Fortification levels (mg/kg)	Recovery (%)	RSD (%)
Glufosinate ammonium	0.1	77.2 ± 3.2	4.1
	0.5	95.5 ± 2.4	2.5
	1	88.9 ± 1.6	1.8

MPP	0.1	98.3 ± 0.4	0.4
	0.5	98.5 ± 0.8	0.8
	1	100.3 ± 1.4	1.4

MPA	0.1	99.3 ± 1.4	1.4
	0.5	99.6 ± 1.3	1.3
	1	99.3 ± 2.0	2.0

## Abbreviations

MPP: 3-Methylphosphinico-propionic acid
MPA: 2-Methylphosphinico-acetic acid
GC: Gas chromatography
FPD: Flame photometric detector
TMOA: Trimethyl orthoacetate
RSD: Relative standard deviations
AMPA: Aminomethylphosphonic acid
CNBF: 4-Chloro-3,5-dinitrobenzotrifluoride
FMOC-Cl: 9-Fluorenylmethylchloroformate
FLD: Fluorescence detection
MSD: Mass-selective detection
LOD: Limit of detection
LC: Liquid chromatography
isoPCF: Isopropyl chloroformate
LOQ: Limit of quantification.
